# Active Children Through Individual Vouchers Evaluation: A Mixed-Method RCT

**DOI:** 10.1016/j.amepre.2019.10.005

**Published:** 2020-02

**Authors:** Michaela L. James, Danielle Christian, Samantha C. Scott, Charlotte E. Todd, Gareth Stratton, Joanne Demmler, Sarah McCoubrey, Julian P.J. Halcox, Suzanne Audrey, Elizabeth A. Ellins, Sinead T. Brophy

**Affiliations:** 1 Swansea University Medical School, Swansea University, Swansea, United Kingdom; 2 Department of Sport and Physical Activity, Edge Hill University, Ormskirk, United Kingdom; 3 College of Engineering, Bay Campus, Swansea University, Swansea, United Kingdom; 4 City and County of Swansea, Swansea, United Kingdom; 5 Population Health Sciences, Bristol, United Kingdom

## Abstract

**Introduction:**

Physical activity declines in adolescence, especially among those in deprived areas. Research suggests this may result from accessibility barriers (e.g., cost and locality). The Active Children Through Individual Vouchers Evaluation RCT aimed to improve the fitness and heart health of teenagers in Wales with the help of teenagers who co-produced the study.

**Study design:**

This study was a mixed-method RCT.

**Setting/participants:**

Before data collection, which took place at baseline, 6 months, and 12 months for both arms, 7 schools were randomized by an external statistician (4 intervention schools, *n*=524; 3 control schools, *n*=385).

**Intervention:**

The Active Children Through Individual Vouchers Evaluation intervention included provision of activity vouchers (£20 per month), a peer mentoring scheme, and support worker engagement for 12 months between January and December 2017. Data analysis occurred February–April 2018.

**Main outcome measures:**

Data included measures of cardiovascular fitness, cardiovascular health (blood pressure and pulse wave analysis), motivation, and focus groups.

**Results:**

The intervention showed a trend to improve the distance ran (primary outcome) and was significant in improving the likelihood of intervention teenagers being fit (OR=1.21, 95% CI=1.07, 1.38, *p*=0.002). There was a reduction in teenagers classified as having high blood pressure (secondary outcome) in the intervention group (baseline, 5.3% [28/524]; 12 months, 2.7% [14/524]). Data on where teenagers used vouchers and evidence from focus groups showed that teenagers wanted to access more unstructured, informal, and social activities in their local areas.

**Conclusions:**

Active Children Through Individual Vouchers Evaluation identified methods that may have a positive impact on cardiovascular fitness, cardiovascular health, and perspectives of activity. Consulting with teenagers, empowering them, and providing more local opportunities for them to take part in activities that are fun, unstructured, and social could positively impact teenage physical activity.

**Trial registration:**

ISRCTN, ISRCTN75594310.

## INTRODUCTION

Being physically active in adolescence is associated with health benefits, including a decreased risk of noncommunicable diseases, such as coronary heart disease and type 2 diabetes,^1^^,^^2^ as well as increased well-being and self-esteem.^3^^,^^4^ Coronary heart disease currently affects more than 7 million people in the United Kingdom.^5^ Therefore, the physical activity (PA) of teenagers is of public health concern.^6^ Public health guidelines recommend 60 minutes of moderate-to-vigorous PA daily.^2^ However, it is reported that only 11% of girls and 20% of boys are sufficiently active in Wales.^7^

Secondary school has been identified as a key period of change in teenagers’ PA behaviors^3^ and is an important setting for promoting PA.^8^ Behaviors adopted during this time are likely to be continued in adulthood.^9^ Teenagers report the main barrier to meeting PA recommendations is accessibility to PA opportunities.^4^^,^^10^^,^^11^ Accessibility is affected by cost, lack of local facilities, and motivation among teenagers,^10^^,^^12^ especially those from disadvantaged backgrounds.^13^

Self-determination theory (SDT)^14^ has emerged as a popular framework for examining motivation and PA^15^ as it differentiates between controlled motivation (e.g., regulated by external pressure or guilt) and autonomous motivation (e.g., regulated by enjoyment).^15^ SDT explains why people engage with, adopt, and maintain PA behaviors.^16^

To overcome accessibility barriers, voucher-based interventions to increase PA in the United Kingdom have been tested previously among adults.^17^^,^^18^ Financial incentives have been effective in increasing PA in adults,^19^, ^20^, ^21^ but it remains uncertain whether a similar approach could work with teenagers.^22^ A mixed-method feasibility study of vouchers has been carried out in 1 school with high levels of deprivation. The vouchers were used to empower teenagers to be consumers and enabled access to activities they normally could not afford.^10^ The teenagers chose to do unstructured, social activities in their local communities. Additional qualitative work identified a disconnect between what teenagers wanted to do and what was available.^11^ This approach was supported by teachers and teenagers who encouraged the development of a larger trial.

The Active Children Through Individual Vouchers Evaluation (ACTIVE) mixed-method RCT^22^ was developed following the feasibility study and subsequent conversations with teenagers recommending what they felt was needed to improve PA opportunities and fitness.^4^ This RCT aimed to assess whether a voucher-based, 12-month, multicomponent intervention can improve the cardiovascular fitness and cardiovascular health of participating teenagers in 4 intervention (compared with 3 control) secondary schools in South Wales.

## METHODS

### Study Population

The ACTIVE RCT was based in secondary schools in South Wales, United Kingdom. All teenagers in Year 9 (aged 13–14 years) were eligible to take part in the study with headteachers granting permission for schools to take part. Randomization occurred before baseline data collection into either intervention or control, with 4 schools assigned to the intervention and 3 schools to the control arm. Control schools were encouraged to continue usual practice and received a mindfulness-based stress reduction course for staff as a thank you for their participation. Schools were not blinded. A detailed protocol has been published.^22^ The College of Human and Health Science Ethics Committee granted ACTIVE ethical approval (reference: 090516).

The RCT was co-produced by teenagers, which allowed some flexibility as teenagers were able to choose how they used their vouchers. SDT was used in the planning of the intervention to understand the reasons teenagers would be likely to engage with the PA provision promoted by the project. Given the empowering nature of the intervention, autonomous motivation was facilitated, giving teenagers a choice rather than being prescriptive. Prescription would be considered controlled motivation. The latter approach has been used in previous studies with mixed, short-term success.^19^^,^^23^ There were no changes to the methods from the protocol after the trial commenced. CONSORT guidelines^24^ informed the analysis and presentation of the study.

Data collection took place at baseline, 6 months, and 12 months for both arms. Baseline collection took place between September to December 2016 with follow-up occurring November 2017 to January 2018. The measures examined for this paper are listed below, and a more detailed explanation including power calculations can be found in the protocol paper.^22^

**Figure 1:**
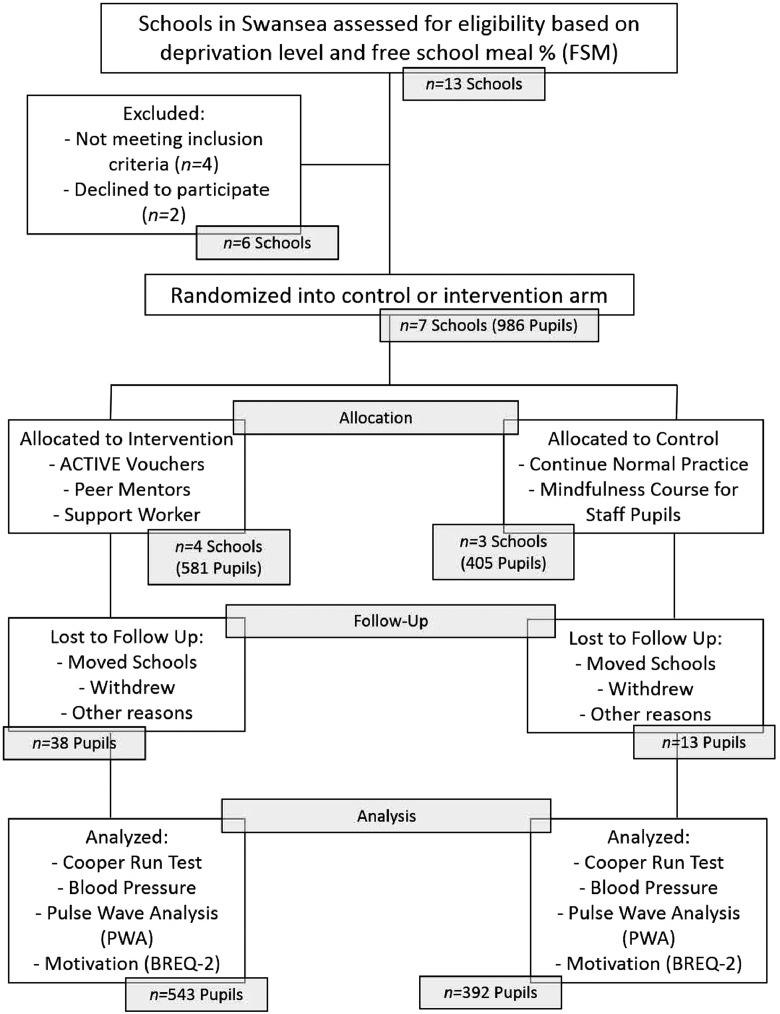
Participant flow of the ACTIVE RCT.

A total of 13 schools were assessed for eligibility; 4 did not meet inclusion criteria of being located in one of Wales’ most deprived areas. School-level deprivation was derived from the Welsh Index of Multiple Deprivation (WIMD), which is used to identify areas of deprivation based on income, employment, health, education, access to services, community safety, environment, and housing.^25^ Schools were coded into 2 groups from this: either more deprived or less deprived for analysis. Two headteachers declined to participate (1 headteacher declined after randomization occurred). This meant 7 secondary schools took part. The demographics of the schools can be seen in Table 1. Figure 1 shows the participant flow of the study; 51 participants were lost from baseline to 12 months (*n*=38 in the intervention) because of moving schools or being absent during testing.

**Table 1:** Consent Rates

School	Pupils in Year 9, total (boys)	Participants in the study, total (boys)	Consent rate, %
School 1	113 (56)	93 (48)	82
School 2	231 (107)	191 (95)	83
School 3	125 (59)	115 (52)	92
School 4	128 (62)	116 (55)	91
School 5	97 (50)	84 (44)	87
School 6	142 (77)	136 (71)	96
School 7	190 (105)	146 (82)	77

ACTIVE, Active Children Through Individual Vouchers Evaluation; BREQ-2, Behavioral Regulation in Exercise Questionnaire; FSM, free school meal; PWA, pulse wave analysis.

Following initial school recruitment, participants (in Year 9, aged 13–14 years) were recruited via school assemblies. Consent was voluntary and involved both written parental consent and pupil assent forms. ACTIVE recruited 909 participants. Members of the local council's sport development team (*n*=15) were also recruited at 12 months for a focus group to assess how partners, who had helped develop the trial, had perceived ACTIVE.

The intervention ran from January to December 2017 and consisted of (1) a voucher scheme, (2) peer mentoring, and (3) support worker engagement. Teenagers received PA vouchers of £20 (4 vouchers in increments of £5) each month for 12 months.^22^ Vouchers could be spent on existing PA provision (e.g., gym membership or sports clubs) or could be used to bring new activities into communities or schools. They could also purchase equipment. How the vouchers were spent was directed by the teenagers.

Teenagers were asked to identify peer mentors (10 from each school) via a peer nomination questionnaire in a similar approach to the Stop Smoking in School Trial.^26^ Their role was to support and encourage the use of vouchers. Mentors had training via formal workshops throughout the school year from the local council and the support worker. An initial training session highlighting the peer mentors’ purpose and developing mentoring skills was provided at the start of the academic year at an external location for the day, and subsequent sessions (1 every half term) took place at the schools lasting 1 hour each. The support worker was based at the University and attended schools to increase pupil awareness of activity provision and encourage teenagers to design new activities through drop-in sessions and school assemblies once a month. All schools received the same amount of contact from the support worker.

The primary aim of this study was to assess the effectiveness of a multicomponent intervention in improving cardiovascular fitness. Secondary aims included evaluating the effects of the intervention on cardiovascular health (as assessed by blood pressure [BP] and pulse wave analysis as an indicator of arterial stiffness), as well as on exercise motivation (using the Behavioral Regulation in Exercise Questionnaire [BREQ-2] and the Relative Autonomy Index).

This study also aimed to explore the qualitative experiences of the intervention from teenagers’ and stakeholders’ perspectives. This helped to provide some further insight into the intervention's effectiveness. The results reported in this paper do not cover all aims from the protocol as some secondary aims will be written as their own standalone papers.

### Measures

All teenagers in Year 9 took part in the Cooper Run Test^27^ in the schools during physical education lessons. This was a 12-minute walk/run test where teenagers were instructed by the research team to complete as many laps of their school's sports hall as possible in the time. Two tests were run during the lesson to allow for peers to record each other's scores. Teenagers were categorized as fit based on whether their total meters ran was considered average and above according to normative data.^28^

Participants had their BP measured using an Omron M2 sphygmomanometer. After resting and sitting down for 5 minutes, participants had 3 measurements taken from their left arm with 2 minutes between each and the average recorded. Participants with systolic BP >130 mmHg were categorized as having high BP. Researchers received training from the University's cardiology department to measure BP.

Pulse wave analysis was used to indicate arterial stiffness using the Vicorder.^29^^,^^30^ Once rested for 5 minutes, participants had 2 measures taken. If both measures of augmentation pressure were within ± 5 mmHg of each other and augmentation index values were within ± 5%, the 2 measures were accepted.^22^ If not, a third was taken, and the mean of all 3 used. Higher augmentation pressure and augmentation index readings can be used as an assessment of arterial stiffness as they indicate cardiovascular disease risk.^31^, ^32^, ^33^

The BREQ-2 questionnaire was used to measure teenagers’ motivation to exercise in relation to their Relative Autonomy Index.^34^ Higher Relative Autonomy Index scores (larger positive weight) indicate greater autonomy to be active, whereas larger negative weights indicate more controlled regulation.^16^ The questionnaire consists of 19 items relating to 5 subscales: amotivation, external regulation, introjected regulation, identified regulation, and intrinsic regulation.^27^ Its validity and reliability has been tested in several populations.^35^, ^36^, ^37^ To gain an insight into the degree of autonomy individuals had for being physically active, the Relative Autonomy Index was calculated; this index has been used in similar studies.^16^^,^^38^ The Relative Autonomy Index is calculated by summing the scores of 5 subscales (3 × intrinsic motivation + 2 × identified regulation − introjected − 2 × external regulation − 3 × amotivation).^16^ The BREQ-2 was chosen because it is accessible for teenagers, clearly written, and uses a Likert scale for responses. Teenagers were asked to complete the questionnaire individually before either the Cooper Run Test or BP measurements.

Digitally recorded semistructured focus groups were conducted at 12 months (*n*=8) by 2 researchers, one who led the conversation and one who made notes to provide feedback to participants as a form of respondent validation.^39^ These consisted of 6–8 teenagers per group, with boys and girls in separate groups, lasting between 20 and 40 minutes, and were conducted at the schools. Participants were selected purposively to gain a variety of viewpoints from those engaging well with the intervention and those whose activity was not based on voucher usage. The aim of the focus groups was to provide a greater understanding of the mediating factors that influenced PA and which aspects of the intervention were successful or unsuccessful.^22^ Members of the local council's community sport development team also took part in a focus group at 12 months to assess how external partners had perceived the intervention. This meant the total number of focus groups was 9.

Deprivation was measured using the WIMD, the official measure of small area deprivation in Wales.^25^ This was done using postcode/lower layer super area output to determine a geographical hierarchy from Welsh Government data. This ranks lower layer super area outputs from most to least deprived.

### Statistical Analysis

Linear mixed effects multilevel regression with intention-to-treat principles was used to analyze the effects of the intervention on the primary outcome in terms of distance. This was clustered by school and at an individual level in Stata, version 12. Logistic regression was also used to assess whether the intervention had an effect on whether pupils were fit or not fit. For secondary outcomes, comparisons were made between baseline and 12 months with differences and CIs presented for a measure of estimation. Data analysis occurred February–April 2018.

Two independent statisticians carried out parallel data analysis on all outcomes to avoid researcher bias. Multiple imputation of missing data because of absence during some testing was conducted using the chained equations^39^ command in Stata. Data for the primary outcome of cardiovascular fitness was imputed for 102 participants at baseline and 170 at follow-up using measures of fitness at other timepoints (baseline/follow-up), sex, and deprivation. This generated one complete data set, which was used for analysis.

Transcription of the focus groups were verbatim, and NVivo, version 10 was used to manage, code, and analyze the data with 2 researchers validating the themes derived from the data via triangulation. Braun and Clarke's Phases of Thematic Analysis^40^ identified and reported codes and themes in the focus groups.

## RESULTS

Baseline characteristics of both the intervention and control groups can be seen in Table 2. There was very little difference in the percentages of boys and girls in both arms. The intervention group was more deprived according to the WIMD but received a lower percentage of free school meals.

**Table 2:** Baseline Characteristics

Characteristic	Control	Intervention
Total, *n* (%)	385 (42)	524 (58)
Sex, *n* (%)		
Boy	213 (55)	254 (48)
Girl	172 (45)	270 (52)
School deprivation		
WIMD, mean	1,156	531
Free school meal %, mean	33	23

WIMD, Welsh Index of Multiple Deprivation.

Regression models were run for the primary outcome (Appendix Table 1, available online) and secondary outcomes (Appendix Table 2, available online). However, for clarity and succinctness, comparison between outcomes for the intervention and control groups can be seen in Table 3.

**Table 3:** Intervention Compared with Control in Terms of Outcomes

Measure	Control	Intervention	Difference
Cooper run (% fit)			
Total, *n*	384	524	
Baseline, % (*n* or 95% CI)	35.9 (138)	33.5 (176)	2.4 (−3.9, 8.6)
12 months, % (*n* or 95% CI)	30.4 (117)	33.0 (173)	−2.6 (−8.6, 3.6)
Difference, % (95% CI)	5.4 (−0.6, 1.6)	0.5 (−4.5, 5.7)	**4.9 (2.7, 7.6)**
Boys, *n*	212	254	
Baseline, % (*n* or 95% CI)	22.1 (47)	24.4 (62)	−2.3 (−9.9, 5.5)
12 months, % (*n* or 95% CI)	18.3 (39)	20.0 (51)	−1.7 (−8.9, 5.5)
Difference, % (95% CI)	3.8 (−3.5, 11.1)	4.4 (−2.1, 10.8)	−0.6 (−0.2, 4.6)
Girls, *n*	172	270	
Baseline, % (*n* or 95% CI)	52.9 (91)	42.2 (114)	**10.7 (11.5, 20.2)**
12 months, % (*n* or 95% CI)	45.3 (78)	45.1 (122)	0.2 (−9.4, 9.7)
Difference, % (95% CI)	7.5 (−2.8, 17.9)	−2.9 (−10.8, 4.8)	**10.4 (0.4, 9.7)**
Deprived, *n*	146	431	
Baseline, % (*n* or 95% CI)	36.3 (53)	35.0 (151)	1.3 (−7.7, 10.2)
12 months, % (*n* or 95% CI)	30.8 (45)	32.4 (140)	−1.6 (−10.4, 7.1)
Difference, % (95% CI)	5.5 (−4.4, 15.4)	2.6 (−3.1, 8.2)	2.9 (−0.4, 7.9)
Not deprived, *n*	238	93	
Baseline, % (*n* or 95% CI)	35.7 (85)	26.8 (25)	8.9 (−2.4, 20.1)
12 months, % (*n* or 95% CI)	30.2 (72)	35.4 (33)	−5.2 (−16.4, 5.9)
Difference, % (95% CI)	5.5 (−2.4, 13.3)	−8.6 (−20.6, 3.4)	**14.1 (4.1, 19.2)**
Cooper run (distance, m)			
Total, *n*	384	524	
Baseline, m (SD or 95% CI)	1,811.8 (±365.5)	1,781.9(±373.5)	29.9 (−18.9, 78.6)
12 months, m (SD or 95% CI)	1,756.0 (±384.4)	1,762.3 (±421.1)	−6.3 (−59.8, 47.2)
Difference, m (95% CI)	55.7 (11.1, 100.3)	19.6 (−16.7, 55.9)	36.1 (−93.1, 20.9)
Boys, *n*	212	254	
Baseline, m (SD or 95% CI)	1,989.9 (±346.0)	2,010.9 (±335.7)	−21 (−83.1, 41.2)
12 months, m (SD or 95% CI)	1,897.1 (±390.7)	1,953.2 (±400.3)	−56.1 (−128.4, 16.3)
Difference, m (95% CI)	92.8 (26.4, 159.1)	57.7 (0.2, 115.2)	35.1 (−122.2, 52.0)
Girls, *n*	172	270	
Baseline, m (SD or 95% CI)	1,592.2 (±252.1)	1,566.5 (±263.1)	25.7 (−23.9, 75.3)
12 months, m (SD or 95% CI)	1,582.1 (±295.8)	1,582.7 (±356.8)	−0.6 (−64.7, 63.5)
Difference, m (95% CI)	10.1 (−46.6, 66.9)	−16.1 (−61.4, 29.0)	26.2 (−98.5, 46.1)
Deprived, *n*	146	431	
Baseline, m (SD or 95% CI)	1,806.2 (±295.4)	1,783.1 (±371.6)	23.1 (−43.4, 89.6)
12 months, m (SD or 95% CI)	1,770.5 (±359.3)	1,763.5 (±412.5)	7 (−68.2, 82.1)
Difference, m (95% CI)	35.7 (−27.9, 99.4)	19.6 (−19.2, 58.4)	16.1 (−92.2, 60.0)
Not deprived, *n*	238	93	
Baseline, m (SD or 95% CI)	1,815.2 (±403.0)	1,776.3 (±384.1)	38.9 (−56.7, 134.6)
12 months, m (SD or 95% CI)	1,747.1 (±363.2)	1,756.5 (±461.1)	−9.4 (−109.8, 91.1)
Difference, m (95% CI)	68.1 (7.3, 128.7)	19.8 (−79.9, 119.5)	48.3 (−163.2, 66.6)
Blood pressure (% high)			
Total, *n*	384	524	
Baseline, % (*n* or 95% CI)	1.6 (6)	5.3 (28)	−3.7 (−5.5, 0.2)
12 months, % (*n* or 95% CI)	3.1 (12)	2.7 (14)	0.4 (−1.7, 2.9)
Difference, % (95% CI)	−1.4 (−3.7, 0.6)	2.6 (−3.0, 5.0)	−4 (−0.9, 3.0)
Boys, *n*	212	254	
Baseline, % (*n* or 95% CI)	2.4 (5)	6.7 (17)	−4.3 (0.4, 8.3)
12 months, % (*n* or 95% CI)	4.2 (9)	3.5 (9)	0.7 (−2.9, 4.3)
Difference, % (95% CI)	−1.8 (−1.7, 5.7)	3.2 (−0.7, 7.2)	−1.4 (−1.9, 4.4)
Girls, *n*	172	270	
Baseline, % (*n* or 95% CI)	**0.6 (1)**	**4.1 (11)**	**−3.5 (0.3, 6.6)**
12 months, % (*n* or 95% CI)	1.7 (3)	1.9 (5)	0.2 (−3.3, 2.7)
Difference, % (95% CI)	−1.1 (−1.7, 4.4)	2.2 (−0.7, 5.4)	−3.3 (−2.1, 3.7)
Deprived, *n*	146	431	
Baseline, % (*n* or 95% CI)	2.0 (3)	4.6 (20)	−2.6 (−1.5, 5.3)
12 months, % (*n* or 95% CI)	3.4 (5)	2.3 (10)	1.1 (−1.6, 5.5)
Difference, % (95% CI)	−1.4 (−2.9, 5.9)	2.3 (−0.1, 4.9)	−3.7 (−2.6, 3.0)
Not deprived, *n*	238	93	
Baseline, % (*n* or 95% CI)	**1.3 (3)**	8.6 (8)	**7.3 (2.5, 14.8)**
12 months, % (*n* or 95% CI)	2.9 (7)	4.3 (4)	−1.4 (−2.6, 7.7)
Difference, % (95% CI)	−1.6 (−1.1, 4.8)	4.3 (−3.2, 12.2)	−5.9 (−1.0, 8.9)
Augmentation pressure (mmHg)			
Total, *n*	384	524	
Baseline, mmHg (SD or 95% CI)	4.9 (±2.5)	5.0 (±2.6)	−0.1 (−0.5, 0.1)
12 months, mmHg (SD or 95% CI)	4.1 (±2.2)	4.0 (±2.4)	0.1 (−0.2, 0.3)
Difference, mmHg (95% CI)	0.8 (0.4, 1.1)	1 (0.7, 1.3)	0.2 (−0.1, 0.7)
Boys, *n*	212	254	
Baseline, mmHg (SD or 95% CI)	4.6 (±2.7)	4.6 (±2.6)	0.0 (−0.4, 0.5)
12 months, mmHg (SD or 95% CI)	4.1 (±2.2)	4.2 (±2.5)	0.1 (−0.4, 0.4)
Difference, mmHg (95% CI)	0.5 (−0.0, 0.9)	0.4 (−0.0, 0.8)	0.1 (−0.7, 0.5)
Girls, *n*	172	270	
Baseline, mmHg (SD or 95% CI)	5.2 (±2.3)	5.5 (±2.4)	−0.3 (−0.7, 0.1)
12 months, mmHg (SD or 95% CI)	4.0 (±2.2)	3.9 (±2.2)	0.1 (−0.3, 0.5)
Difference, mmHg (95% CI)	1.2 (0.7, 1.7)	1.6 (1.2, 2.0)	0.1 (−0.1, 0.9)
Deprived, *n*	146	431	
Baseline, mmHg (SD or 95% CI)	4.5 (±3.2)	5.2 (±2.4)	**−0.7 (−1.2, −0.1)**
12 months, mmHg (SD or 95% CI)	4.0 (±2.0)	4.1 (±2.3)	−1 (−0.5, 0.3)
Difference, mmHg (95% CI)	0.5 (−0.1, 1.1)	1.3 (0.8, 1.4)	**0.8 (0.1, 1.4)**
Not deprived, *n*	238	93	
Baseline, mmHg (SD or 95% CI)	5.1 (±2.0)	4.2 (±2.9)	**0.9 (0.3, 1.4)**
12 months, mmHg (SD or 95% CI)	4.1 (±2.3)	3.6 (±2.6)	0.5 (−0.0, 1.0)
Difference, mmHg (95% CI)	1.0 (0.5, 1.3)	0.6 (−0.2, 1.3)	0.4 (−1.2, 0.4)
Augmentation index (%)			
Total, *n*	384	524	
Baseline, % (SD or 95% CI)	9.5 (±4.0)	10.0 (±4.6)	**−0.5 (−1.1, 0.0)**
12 months, % (SD or 95% CI)	7.4 (±3.2)	7.6 (±4.3)	−0.2 (−0.6, 0.3)
Difference, % (95% CI)	2.1 (1.5, 2.5)	2.4 (1.8, 2.9)	−0.3 (−0.4, 1.0)
Boys, *n*	212	254	
Baseline, % (SD or 95% CI)	8.8 (±3.9)	9.1 (±4.8)	−0.3 (−1.1, 0.5)
12 months, % (SD or 95% CI)	7.9 (±3.1)	8.1 (±4.5)	−0.2 (−0.9, 0.5)
Difference, % (95% CI)	0.9 (0.1, 1.5)	1.0 (0.2, 1.7)	−0.1 (−0.9, 1.1)
Girls, *n*	172	270	
Baseline, % (SD or 95% CI)	10.3 (±3.9)	10.9 (±4.1)	−0.6 (−1.3, 0.2)
12 months, % (SD or 95% CI)	6.9 (±3.2)	7.2 (±4.0)	−0.3 (−1.0, 0.4)
Difference, % (95% CI)	3.4 (2.6, 4.2)	3.7 (3.0, 4.4)	−0.3 (−0.7, 1.3)
Deprived, *n*	146	431	
Baseline, % (SD or 95% CI)	8.8 (±4.5)	10.4 (±4.3)	**−1.6 (−2.4, −0.8)**
12 months, % (SD or 95% CI)	7.6 (±3.0)	7.8 (±3.9)	−0.2 (−0.8, 0.5)
Difference, % (95% CI)	1.2 (0.2, 2.1)	2.6 (2.0, 3.1)	**−1.4 (−2.5, −0.4)**
Not deprived, *n*	238	93	
Baseline, % (SD or 95% CI)	9.9 (±3.6)	8.3 (±5.2)	**1.6 (0.6, 2.6)**
12 months, % (SD or 95% CI)	7.4 (±3.3)	7.0 (±5.5)	0.4 (−0.5, 1.3)
Difference, % (95% CI)	2.5 (1.9, 3.2)	1.3 (−0.2, 2.8)	1.2 (−0.1, 2.5)
Motivation, BREQ-2 (% autonomous RAI)			
Total, *n*	384	524	
Baseline, % (*n* or 95% CI)	98.1 (378)	97.1 (509)	1 (−0.9, 3)
12 months, % (*n* or 95% CI)	97.9 (377)	97.9 (513)	0 (−1.8, 1.9)
Difference, % (95% CI)	0.2 (−1.5, 2.1)	−0.8 (95–2.4, 0.9)	0.9 (−0.7, 1.7)
Boys, *n*	212	254	
Baseline, % (*n* or 95% CI)	98.1 (209)	97.6 (248)	0.5 (−2.1, 3.1)
12 months, % (*n* or 95% CI)	98.5 (210)	97.6 (248)	0.9 (−1.5, 3.4)
Difference, % (95% CI)	−0.4 (−2.9, 1.9)	0 (−2.1, 2.1)	−0.4 (−1.0, 2.6)
Girls, *n*	172	270	
Baseline, % (*n* or 95% CI)	98.2 (169)	96.6 (261)	0.6 (−1.5, 4.7)
12 months, % (*n* or 95% CI)	97.0 (167)	98.1 (265)	−1.1 (−3.9, 1.8)
Difference, % (95% CI)	1.2 (−1.6, 3.9)	−1.5 (−4.0, 1.0)	2.7 (−3.2, 3.0)
Deprived, *n*	146	431	
Baseline, % (*n* or 95% CI)	97.2 (143)	98.1 (423)	−0.9 (−3.5, 1.8)
12 months, % (*n* or 95% CI)	99.3 (146)	98.1 (423)	1.2 (−1.1, 3.5)
Difference, % (95% CI)	−2.1 (−5.0, 0.9)	0 (−1.5, 1.5)	**2.1 (0.4, 5.8)**
Not deprived, *n*	238	93	
Baseline, % (*n* or 95% CI)	98.7 (235)	92.4 (86)	**6.3 (2.1, 10.3)**
12 months, % (*n* or 95% CI)	97.0 (231)	96.7 (90)	3 (−3.8, 4.4)
Difference, % (95% CI)	1.7 (−0.6, 4.0)	−4.3 (−10.3, 17.0)	6 (−1.0, 8.9)
Motivation (total RAI)			
Total, *n*	384	524	
Baseline, RAI score (SD or 95% CI)	9.1 (±3.5)	9.2 (±4.2)	−0.1 (−0.6, 0.4)
12 months, RAI score (SD or 95% CI)	8.6 (±3.2)	8.8 (±3.4)	−0.2 (−0.6, 0.2)
Difference, RAI score (95% CI)	**0.5 (0.1, 1.0)**	**0.4 (0.0, 0.8)**	0.1 (−0.2, 0.6)
Boys, RAI score, *n*	212	254	
Baseline, RAI score (SD or 95% CI)	9.3 (±3.5)	9.4 (±4.2)	−0.1 (−0.8, 0.5)
12 months, RAI score (SD or 95% CI)	8.8 (±3.0)	9.2 (±3.5)	−0.4 (−1.0, 0.1)
Difference, RAI score (95% CI)	0.4 (−0.1, 1.1)	0.2 (−0.3, 0.8)	0.2 (−0.1, 1.0)
Girls, RAI score, *n*	172	270	
Baseline, RAI score (SD or 95% CI)	8.9 (±3.4)	9.0 (±4.2)	−0.1 (−0.8, 0.6)
12 months, RAI score (SD or 95% CI)	8.2 (±3.5)	8.4 (±3.2)	−0.1 (−0.7, 0.5)
Difference, RAI score (95% CI)	0.7 (−0.0, 1.3)	**0.6 (0.0, 1.1)**	0.1 (−0.5, 0.7)
Deprived, RAI score, *n*	146	431	
Baseline, RAI score (SD or 95% CI)	8.7 (±3.8)	9.4 (±3.7)	−0.7 (−1.4, 0.0)
12 months, RAI score (SD or 95% CI)	8.8 (±3.1)	8.8 (±3.4)	−0.0 (−0.6, 0.5)
Difference, RAI score (95% CI)	−0.1 (−0.7, 0.6)	**0.6 (0.1, 0.9)**	−0.7 (−5.6, 6.9)
Not deprived, RAI score, *n*	238	93	
Baseline, RAI score (SD or 95% CI)	9.4 (±3.3)	8.4 (±6.0)	1.0 (−0.0, 1.9)
12 months, RAI score (SD or 95% CI)	8.4 (±3.3)	8.5 (±3.4)	−0.1 (−0.9, 0.6)
Difference, RAI score (95% CI)	**1.0 (0.3, 1.5)**	−0.1 (−1.3, 1.0)	1.1 (−6.9, 9.4)

*Note:* Boldface indicates statistical significance (*p*<0.05).

BREQ-2, Behavioral Regulation in Exercise Questionnaire; RAI, Relative Autonomy Index.

The 6-month data showed the same observed trends for the outcomes. However, for clarity, comparisons have been presented between baseline and 12 months.

Linear mixed effects multilevel regression (Appendix Table 1, available online) showed that, overall, the intervention group ran fewer meters than the control group. However, the interaction between group and time (being in the intervention at 12 months) showed a trend that the intervention improved the distance run by teenagers, although this was not statistically significant. Therefore, the intervention group showed a trend to run farther than the control group at 12 months.

The number of teenagers categorized as fit in the control group declined by 5.4%, but there was only a reduction of 0.6% in the intervention group at 12 months. Logistic regression showed significantly higher odds of being fit at 12 months in the intervention group than the control group (OR=1.21, 95% CI=1.07, 1.38, *p*=0.002) (Appendix Table 1, available online). Girls in the intervention group showed a trend to become fitter (3% more children were fit) and the girls in the control group became less fit (7.5% more children were unfit).

The proportion of participants with high BP in the intervention group fell (baseline, 5.3% [28/524]; 12 months, 2.7% [14/524]), whereas the proportion of participants with high BP in the control increased (baseline, 1.6% [6/384]; 12 months, 3.1% [12/384]). Augmentation pressure and augmentation index improved in both arms. Deprived children in the intervention group saw a significant decrease in augmentation pressure compared with the control group.

Participants were autonomously motivated, with between 96% and 99% of teenagers in both arms autonomously motivated at baseline and 12 months. Total motivation showed a decreasing trend between the 2 timepoints. Girls in the intervention showed a significant decrease (0.6, 95% CI=0.0, 1.1), as well as deprived teenagers (0.6, 95% CI=0.0, 1.1). However, this change in the mean did not impact the percentage of participants who were autonomously motivated in the intervention.

Trampolining accounted for almost half of the voucher usage (49.1%), followed by laser tag (11.5%) and the waterpark (slides and surfing, 7.3%) (Appendix Table 3, available online). ACTIVE helped set up lunchtime clubs in 2 different schools at the request of teenagers, an unstructured football session, dance, and parkour.

The participants used 26.2% (7,545/28,800) of all the vouchers available, and boys made up 52% of the vouchers spent. Common themes for not using the vouchers included the lack of local provision (Appendix Table 4, available online).

Focus group conversations about the impact of the intervention flowed around 2 themes, the breaking down of cost barriers and changes in perception of PA. Teenagers reported they no longer had to ask their parents for money, which had often been a barrier. One boy said he was able to “go places and do different things” because of knowing more about what was available. The local council echoed this, they felt “…they [the vouchers] are making people more aware of what is in the local area so that they can be active…”

Teenagers reported changes in their local area thanks to the vouchers. A leisure center doubled the value of the vouchers to £10: “…they've doubled up, so like one is now worth £10 because it costs more than £5 for some of the sessions” (girl). In addition to this, the local trampoline park added free food, which made PA feel more social and welcoming. One girl stated, “…because then like you go there, you have food as well, it's like more of a thing.”

Some participants noted that the study had changed their view of PA. For example, one girl noted, “you don't realize its exercise…” For girls, this change of definition seemed to be very helpful as “you don't have to wear sports clothes, and it doesn't matter, but you can make a day of it, so like you can go to town, and then maybe go to Laser Zone” (girl). Changes in the perception were also present in the council focus group who discussed using ACTIVE's data to change their approach to PA provision. One individual said they would “use the results to shape and inform our planning for our areas” as well as tackling the issues associated with teenage inactivity. They noted that the data could underpin “how we can address them [barriers identified in ACTIVE] within our [school and community] programs.”

There were some aspects of the study that teenagers said were less positive. There was a lack of clarity about who the peer mentors were, and one boy stated “…like I haven't felt the need to like go to one.” The participants suggested that the mentors should be chosen differently: “get a gym teacher to look at who does most sports in the school and like who enjoys it most” (boy). One girl said, “I feel like some of the people that have been chosen don't really want to be involved...” and a different selection process may have protected against this.

The teenagers thought that the support worker was beneficial, as they created awareness of what was new or “if anything had changed, which was really informative and nice” (boy, Focus Group 1). However, some teenagers noted that the timings of the support worker were not ideal; in particular, they said morning assemblies were a time when they do not pay attention. Additionally, the local council noted that one intervention school was a Welsh-medium school to which teenagers were likely to have commuted; therefore, the participants would not have benefited from any change in provision around the school.

## DISCUSSION

Despite teenagers in the intervention running fewer meters than the control schools at both timepoints, being in the intervention showed trends of improving the distance run and was significant in improving the number of girls classified as fit. This is novel, as it provides evidence that giving teenagers a choice to access unstructured activities can benefit fitness. It is also novel that this study has shown the intervention helped reduce the number of teenagers that had high BP. Teenagers were autonomously motivated^14^ even before the implementation of the intervention. This provides a rationale, in line with SDT, as to why teenagers chose to access enjoyable, fun, and social activities on the project rather than more prescriptive, structured forms of PA.

There was some evidence that the intervention decreased autonomous motivation for girls and more deprived teenagers. This could be because the vouchers were perceived as an external pressure to be active.^16^ However, the increase in fitness suggests that incentives could be beneficial in the short term while young people explore activity opportunities or used longer term in groups with particularly low fitness. Ultimately, this study highlights that lack of motivation is not the issue; young people do not need to be pressured into being active. What is currently on offer for teenagers is not what they want. The vouchers gave a novel insight into what teenagers enjoy doing and provided evidence that there should be a focus on listening to teenagers^4^^,^^11^ as opposed to being prescriptive. Activity providers should consult with teenagers to overcome accessibility barriers.

The study highlights some important baseline findings regarding teenage PA. Namely, approximately 65% of children are unfit and this may increase by 5% per year judging by changes in the control group. This is in line with previous findings.^3^^,^^7^^,^^41^ However, listening to what teenagers want and helping them overcome accessibility issues could go some way to preventing this. Previous PA interventions have been prescriptive, with specific activities or teaching strategies given to teenagers.^3^^,^^23^^,^^38^^,^^42^ They have had mixed success to date and are often short term.^19^^,^^23^ The findings from ACTIVE suggest there is a need to focus on allowing teenagers to have a say in activity provision.

The way the vouchers were used showed that unstructured fun activities were favored, which supports findings that teenagers see structured activities as a barrier to being active.^4^^,^^10^ The popularity of the trampoline park suggests that activities that place emphasis on fun are more appealing to teenagers.^4^^,^^11^ This is not to say that trampoline parks are a generalizable provision, but rather the nontraditional and social aspects are elements to implement in the future.

This study provides evidence that a co-produced intervention can have wider community benefits. Teenagers said that there had been changes made to local PA provision; one participant said that cost had been altered in a positive way. In addition, the local council used the feedback from ACTIVE to underpin their future planning. This provides evidence of the sustainability of ACTIVE as it helped inform the delivery of community- and school-based PA for teenagers.

Some barriers still existed that the intervention did not overcome. Participants used 26.2% of the vouchers. Some accessibility barriers were still present^43^; teenagers stated they found it difficult to travel to activities further away from their homes. This suggests there is a lack of services and facilities in deprived areas and inability to travel for deprived children. Therefore, more research is needed to develop ways of overcoming this issue, whether addressing the transport itself or bringing more activities to local communities. Some community activities had either an upper age limit of 10 years or a minimum age limit of 16 years, and there was little specifically available and advertised for those aged 11–16 years. The high percentage of autonomously motivated teenagers may also have contributed to low voucher use as they may have perceived the vouchers as an external pressure.

The peer support aspect of the intervention was unsuccessful despite seeing success in previous interventions.^44^ Participants noted that the selected individuals were unapproachable, and teenagers would not really ask a peer for advice, help with being active, or take advice on things to do. Care needs to be taken during the selection process of peer mentors. Again, this complements findings that teenagers were autonomously motivated^14^ and did not need an external influence to get them more active.

### Limitations

Baseline data collection occurred after randomization, and given the nature of the study, participants were not blinded. This study was only able to measure outcomes of teenagers who consented, and they may have been more motivated and interested in being active. Owing to school schedules, there was overlap where some teenagers were still receiving vouchers during the follow-up testing (47%, *n*=430). This may have influenced some measures. There was also no follow-up after the vouchers stopped, which could have provided evidence of the long-term impact of ACTIVE.

This study was unable to assess whether the vouchers substituted previous activities with more fun activities or added additional PA to the teenagers’ weeks. It is unknown if the low voucher use reflects that some students were already very active or if higher voucher use contributed to better outcomes. This is a limitation that could be addressed in future research.

ACTIVE selected deprived schools if they were in a deprived area. However, for one school at least, this was not a good method of identifying deprivation of teenagers. Future work could use free school meal percentage.

## CONCLUSIONS

The ACTIVE intervention provides evidence that to improve fitness, health, and perceptions of PA, there should be a focus on listening to teenagers and providing more local opportunities to take part in activities that are fun, unstructured, and social to make a real difference to teenage PA. Future interventions should focus on advocating and empowering teenagers so that PA opportunities are what they want and not what adults think they should have.

## Appendix SUPPLEMENTAL MATERIAL

Image, application 1

## Footnotes

Supplemental materials associated with this article can be found in the online version at https://doi.org/10.1016/j.amepre.2019.10.005.
