# Supplementary material for: Active Children Through Individual Vouchers Evaluation: A Mixed-Method RCT
Source: Am J Prev Med. 2020 Feb;58(2):232–43. doi: 10.1016/j.amepre.2019.10.005 (PMC6955646; doi:10.1016/j.amepre.2019.10.005)
Supplement: Supplementary file 1 [file mmc1.pdf]

Appendix Table 1. Outcomes of Regression Models

**Covariate**	**Slope**	**CI**
Mixed effects multi-level regression (distance ran)		
Group (intervention)	–32.3	–89.0, 24.4
Time (12-month follow-up)	–55.3	–98.3, –12.1
Intervention **X** time (slope difference of intervention versus control at 12 months)	35.6	–21.2, 92.4
Logistic regression (1=fit/0=not fit)		
Group (intervention)	1.21	1.07, 1.38

Appendix Table 2. Outcomes of Regression Models for Secondary Outcomes

**Outcome**	**Slope**	**CI**
Blood pressure (%), intervention	–0.13	**–0.21, –0.05**
Blood pressure (mmHg), intervention	–1.4	–3.0, 0.08
Augmentation pressure (mmHg), intervention	0.02	–0.08, 0.13
Augmentation index (%), intervention	–0.04	–0.30, 0.21
Motivation (%), intervention	–0.07	–1.49, 1.35
Motivation (total), intervention	0.14	–0.36, 0.65

Note: Boldface indicates statistical significance ($p<0.05$).

Appendix Table 3. Frequency of Voucher Use Stratified by Gender

Activity	Total	Girls	Boys
Aqua aerobics	3	0	3
Aqua zumba	2	2	0
Badminton	13	5	8
Boxing equipment	13	0	13
Cycling equipment	361	76	285
Miscellaneous equipment	37	36	1
Equipment for school	19	0	19
Equipment from Nash Sport	34	7	27
Fitness equipment	368	187	154
Foot golf	69	19	50
Football	407	7	400
Football equipment	122	10	112
Gym membership	357	182	175
Gym pay & play	211	134	77
Gymnastics	4	3	1
Court hire	4	0	4
Kickboxing	3	0	3
Laser tag	862	514	348
Martial arts	6	6	0
Martial arts equipment	55	28	27
Paintballing	4	4	0
Parkour	48	0	48
Play area	3	2	1
Rock climbing	10	10	0
Skateboard equipment	94	62	32
Skateboarding	23	0	23
Swimming	48	20	28
Swimming equipment	77	71	6
Tennis	8	0	8
Tennis equipment	13	0	13
Trampolining	3,692	1,914	1,778
Water park	547	288	253
Zumba	3	3	0

Appendix Table 4. Reasons for Lack of Voucher Use

Theme	Representative quote
There was nothing to do in the area.	"I can't really think of many places you could use them, we haven't got that many places in the area." (Girl)
It required travel to get to PA places.	"Sometimes it's hard to get to, you know like the paintballing place, people find it hard to get to places like that, like buses and stuff." (Girl)
Participants were too busy.	"School work and that, you don't get time to do anything," (Boy) and, "It's just very busy now, especially with the GCSE period." (Girl)
Participants were not interested in being active.	"Like, they don't see the point," (Boy) and, "Because of Xboxes that people are using, they're too busy focused on social media." (Girl)

PA, physical activity.
